# Epicardial adipose tissue radiomic features from pre-procedural CT to predict atrial fibrillation recurrence after catheter ablation for pulmonary vein isolation

**DOI:** 10.3389/fcvm.2026.1765419

**Published:** 2026-02-26

**Authors:** Guoxiang Ma, Shuai Shang, Zhen Bao, Hui Liu, Huling Li, Kai Wang, Baopeng Tang, Yanmei Lu

**Affiliations:** 1 College of Medical Engineering and Technology, Xinjiang Medical University, Urumqi, China; 2 Department of Cardiac Pacing and Electrophysiology, The First Affiliated Hospital of Xinjiang Medical University, Urumqi, China; 3 Xinjiang Key Laboratory of Cardiac Electrophysiology and Remodeling, The First Affiliated Hospital of Xinjiang Medical University, Urumqi, China; 4 Postdoctoral Research Station of Public Health and Preventive Medicine, School of Public Health, Xinjiang Medical University, Urumqi, China

**Keywords:** atrial fibrillation, epicardial adipose tissue, machine learning, pulmonary vein isolation, radiomics

## Abstract

**Purpose:**

This study aimed to develop and validate a machine learning model that integrates radiomic features of epicardial adipose tissue (EAT) from pre-procedural CT angiography with clinical variables to predict atrial fibrillation (AF) recurrence after pulmonary vein isolation (PVI).

**Materials and methods:**

This retrospective study initially included 1,551 AF patients who underwent PVI. After data integrity screening and 1:1 propensity score matching (PSM) to balance confounding factors, the final analysis cohort consisted of 302 patients (151 with recurrence and 151 without recurrence). EAT was segmented from preoperative CT angiography images using a SwinUNETR model, which was pre-trained via transfer learning on manually annotated images. Following segmentation, radiomic features were extracted. Subsequently, six machine learning models were developed and evaluated.

**Results:**

The SwinUNETR segmentation model achieved a dice similarity coefficient of 0.87. For AF recurrence prediction, the fusion model demonstrated superior and robust performance in internal validation. The random forest-based fusion model achieved the highest area under the curve (AUC) of 0.81 (95% CI: 0.59–0.87). Key predictive features included NT-proBNP and texture heterogeneity features from EAT, which align with known pathophysiological mechanisms involving systemic inflammation, metabolic dysregulation, and local atrial adipose tissue remodeling.

**Conclusion:**

A fusion model incorporating EAT radiomics and clinical variables effectively predicts AF recurrence after PVI, with ensemble methods showing optimal performance. This study provides a multiscale, interpretable computational tool for individualized postoperative risk stratification, highlighting the complementary role of EAT imaging biomarkers to systemic clinical factors.

## 1 Introduction

Atrial fibrillation (AF) is the most common arrhythmia disorder, which can cause diseases like stroke, heart failure, etc., and significantly increase the risks of death, stroke, heart failure, and cognitive impairment, posing great impact on the patient's quality of life and health (1). In recent years, a series of breakthroughs have been made in predicting AF risk, screening diagnosis, stroke prevention, rhythm control, and catheter ablation (2, 3). Circumferential pulmonary vein isolation (PVI) is a fundamental strategy in catheter ablation for AF. Its core pathophysiological basis lies in the fact that the pulmonary veins and their vestibular regions are the primary origin points of ectopic electrical activities that trigger AF (4). Therefore, this procedure aims to achieve electrical isolation of the pulmonary veins by creating continuous transmural lesions in the vestibular region of the pulmonary veins, ultimately achieving the therapeutic goal of restoring and maintaining sinus rhythm for a long term. However, Postoperative recurrence remains a significant clinical challenge, affecting a significant proportion of patients (5, 6). Studies have shown that almost half of the patients have a recurrence within 1 year after cardioversion, and most recurrences occur within 1 month after PVI (7). However, in the range of 50%–75%, without good predictive strategies to identify patients who are unlikely to benefit from AF ablation *a priori* (8).

Clinical scores have been developed to predict success after PVI. The majority of the clinical models have an AUC of 0.55–0.65, with rare models reaching AUC of 0.75 (9, 10). Most of these predictive scores have not incorporated imaging directly as an input, but included derived measurements from imaging studies such as left atrial size or sphericity index. In recent years, attention has shifted from the cardiac chambers to peripheral epicardial adipose tissue (EAT) as a key regulator of the atrial matrix (11–13). EAT is a visceral fat depot located between the myocardium and pericardium, which is not a passive fat storage but a biologically active endocrine and paracrine organ. In conditions such as obesity and metabolic syndrome, EAT undergoes a phenotypic switch characterized by increased secretion of proinflammatory cytokines, profibrotic factors, and adipokines (14). These bioactive molecules can directly infiltrate the adjacent atrial myocardium, promoting a pro-arrhythmic milieu through localized inflammation, oxidative stress, interstitial fibrosis, and disruption of electrophysiological stability. Consequently, the volume and, more importantly, the qualitative characteristics of EAT have been implicated in the pathogenesis and perpetuation of AF. Accumulating evidence suggests that a larger EAT burden or a stronger inflammatory phenotype is associated with increased risk for AF initiation and recurrence after catheter ablation (15, 16). Therefore, precise quantification of EAT, especially by assessing its radiological characteristics beyond its simple volume, is expected to improve preoperative risk stratification. Investigating the role of EAT in AF recurrence is not only crucial for elucidating the underlying pathophysiological mechanisms but also for developing novel imaging biomarkers to identify patients who would most benefit from ablation therapy and who might require adjunctive treatment strategies.

The integration of deep learning and radiomics has demonstrated considerable potential in medical research, particularly in cardiovascular disease prediction (17–19). Deep learning is good at automatically extracting features from large datasets, while radiomics can perform high-throughput quantification of medical images to reveal disease characteristics. However, the clinical translation of these approaches is often hampered by their dependency on large, well-annotated datasets. To address this issue, we collected retrospective data on 1,551 patients with AF who underwent PVI. Furthermore, to overcome the difficulty of labeling EAT on CT images, we implemented SwinUNETR segmentation model to facilitate efficient and accurate image analysis. In conclusion, this study aimed to develop and validate a combined radiomics and clinical model for predicting postoperative recurrence of PVI. Our work aims to identify potential EAT imaging biomarkers to contribute to more individualized treatment strategies for AF, while also promoting the application of artificial intelligence in cardiovascular medicine.

## 2 Materials and methods

**Figure 1:**
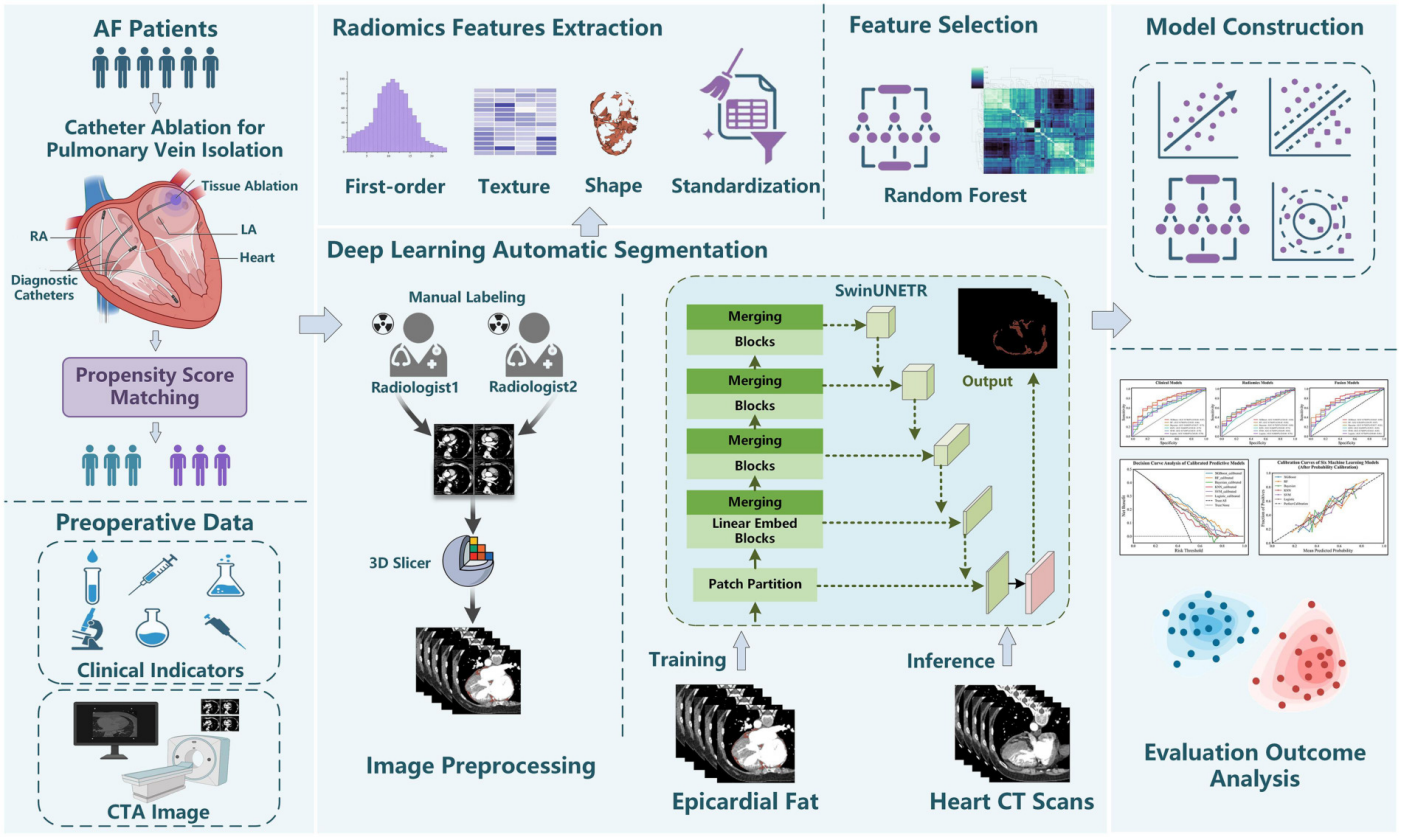
Schematic flowchart of the study design for predicting AF recurrence after catheter ablation for PVI.

The experimental code for this study was written in Python (version 3.9.7) and R (version 4.2.3). The deep learning framework used was MONAI (version 1.3.0; https://monai.io/), a freely available, community-supported frame work specifically designed for medical imaging research. The models were trained on a server equipped with an NVIDIA A800 GPU. The final results of the experiments were evaluated using 10-fold cross-validation. The overall study design and analytical pipeline are schematically illustrated in Figure 1.

### 2.1 Data

**Figure 2:**
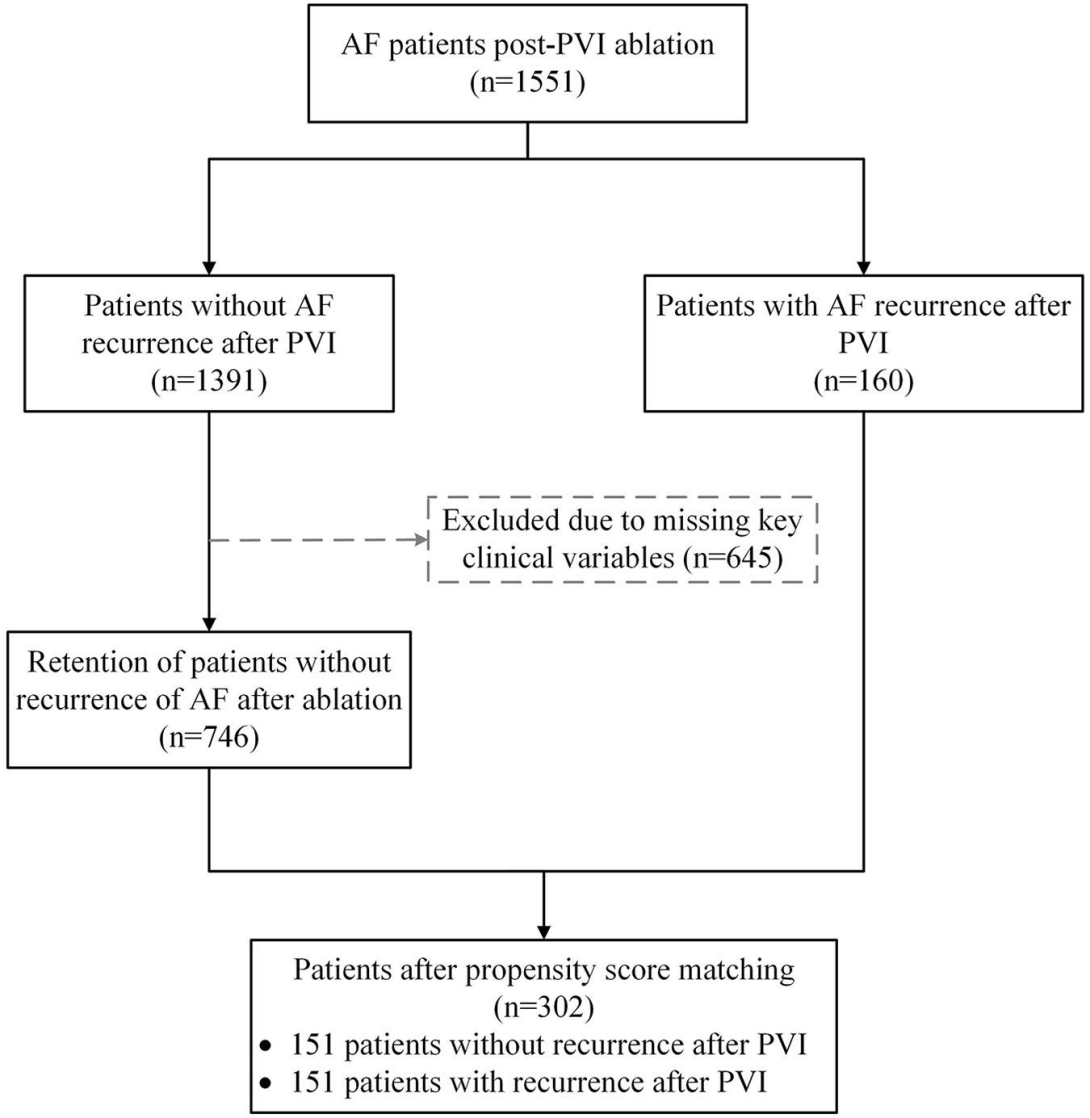
Flowchart of the study patients, and subsequent PSM before statistical analyses.

This study involved 1,551 patients who underwent PVI in the Cardiac Pacing and Electrophysiology Department of the First Affiliated Hospital of Xinjiang Medical University. The definition of AF recurrence is that, 3 months after ablation surgery, any episode of AF, atrial flutter, or atrial tachycardia lasting for more than 30 s is recorded through electrocardiogram monitoring. All patients in the non-recurrence group received at least 6 months of follow-up, during which no documented recurrence of arrhythmia was observed. The patient data included clinical examination data of the first preoperative examination and CTA imaging data of the left atrium and pulmonary vein. Of note, all patients at this center undergo routine imaging before AF ablation procedure, not leading to a substantial selection bias. Detailed inclusion/exclusion criteria and enrollment process are illustrated in Figure 2. Among all eligible cases, 160 patients had a recurrence of AF after catheter ablation for PVI. In contrast, 1,391 patients did not experience recurrence. To ensure data integrity and mitigate potential confounding biases, we applied predefined exclusion criteria. A total of 645 cases were excluded due to incomplete or missing key clinical variables. To evaluate whether this exclusion introduced systematic differences, we compared the available baseline characteristics between the primary cohort (*n* = 1,551) and those retained in the prematching cohort (*n* = 906). The comparison results showed no statistically significant differences between the two groups on most available variables, indicating that the exclusions were primarily driven by data completeness rather than specific clinical phenotypes, This mitigates, to some extent, the selection bias introduced by the exclusion process. Detailed comparison results are provided in Supplementary Table S1. Following this data quality control step, 746 patients without recurrence were retained for subsequent analysis. To enhance the comparability between the recurrence and non-recurrence groups, a 1:1 propensity score matching (PSM) was performed. Matching was based on relevant preoperative clinical characteristics. This process yielded a final well-balanced study population of 302 patients, comprising 151 with AF recurrence and 151 without.

### 2.2 Propensity score matching

In this study, propensity scores were estimated using a multivariate logistic regression model, and the nearest neighbor method was adopted for matching. To minimize potential confounding biases and enhance the comparability between groups, a 1:1 matching was performed between patients with recurrent AF requiring repeat ablation and those without recurrence. This score is defined as the conditional probability of an individual being assigned to a specific group under the condition of a given set of baseline covariates. Then, based on this score, matching is performed between the recurrence group and the non-recurrence group to simulate the effect of a randomized controlled trials, and a cohort with balanced distribution of key baseline characteristics and stronger comparability is constructed. To prevent information leakage from correlated matched pairs during cross-validation, the propensity-matched pairs were kept intact and assigned as a single unit to each fold. Matching was performed using the MatchIt package in R and the following covariates were included for stratification: gender, age, body mass index (BMI), vital signs (body temperature and respiratory rate), smoking status, alcohol use, history of diabetes, hypertension, and surgical history. The balance of baseline characteristics between the matched cohorts was assessed using the standardized mean difference (SMD), with an SMD < 0.1 indicating adequate balance. Finally, the predictive performance of the model was assessed in the matched cohort (20, 21).

### 2.3 Image segmentation of EAT

The CT image data used in the study were collected according to the routine contrast-enhanced imaging protocol. The scan extended from the level of the main pulmonary artery to approximately 2 cm below the diaphragmatic surface of the heart. Before imaging, all patients underwent uniform breathing training. A contrast agent (Ioversol, 350 mg/mL) was administered intravenously via the antecubital vein at a dose of 80–100 mL and an injection rate of 4–5 mL/s. This was followed by a 30 mL saline flush at a flow rate of 4 mL/s.

Medical radiomics research highly relies on accurate labeling data. However, the traditional manual labeling method has inherent limitations such as low efficiency, high cost and poor consistency. In order to break through this bottleneck, a semi-automatic annotation method was developed. Its core value lies in the following: the initial contour is generated with the assistance of algorithms, which significantly improves the annotation efficiency and reduces the labor cost. At the same time, this method can effectively standardize the labeling process, enhance the consistency and repeatability of the data, and provide key technical support for the construction of high-quality data sets and the acceleration of radiomics model development and clinical transformation. To address the challenges of manual annotation, we implemented the SwinUNETR model for semi-automatic segmentation (22). Its integration of a shifted window transformer mechanism allows it to effectively capture global contextual information, leading to improved accuracy and robustness in segmenting complex structures from 3D CT scans. This model was trained on a manually annotated dataset comprising 50 patients. The average number of images per case is 218, for a total of 10,900 images. To generate a high-confidence ground truth for model training, the image annotations were performed through a collaborative consensus process by two radiologists with 6 years of experience, using 3D Slicer (version 5.2.2). A standardized protocol was strictly followed: the delineation plane extended from the lower margin of the right pulmonary trunk to the cardiac apex, with a consistent fat attenuation threshold defined as −190 to −30 HU. During the annotation sessions, the radiologists worked jointly to delineate each case, discussing and resolving any ambiguities regarding EAT boundaries in real time to reach a consensus contour. To further ensure the quality and consistency of the final dataset, all consensus segmentations underwent a final review by a senior cardiothoracic imaging specialist. This rigorous, consensus-driven approach was adopted to prioritize the creation of a single, reliable “gold standard” segmentation per patient, which is crucial for the supervised training of the deep learning model. MONAI frameworks were utilized for model training, with the training set and validation set divided at a ratio of 4:1. The manually annotated dataset comprising 50 patient scans was divided in a stratified manner, with 40 scans allocated for training and 10 scans held out as an independent validation set. This validation set was strictly reserved for the final evaluation of the trained model's segmentation performance and was not used during any stage of model training or hyperparameter tuning. Before model training, spatial transformations, intensity transformations, and rotational transformations were applied to the original 3D images for image augmentation. During model training, the image size was 512 × 512 × 512, the batch size was set to 2, the patch resolution was 96 × 96 × 96, the initial learning rate was 0.0001, and 10,000 iterations were performed using the AdamW optimizer. The pre-trained model was used to initialize the backbone network's weights. The segmentation performance of the final model was evaluated using the dice similarity coefficient (DSC) to assess volumetric overlap and the hausdorff distance (HD) to quantify boundary accuracy.

### 2.4 Radiomics features extraction and standardization

Radiomics facilitates the translation of medical images into a high-dimensional, quantitative feature space. By analyzing the intensity, shape, and texture patterns within a region of interest (ROI), it provides a comprehensive characterization of tissue heterogeneity for subsequent data mining. To ensure the reproducibility and standardization of our radiomics analysis in compliance with the image biomarker standardisation initiative (IBSI) guidelines, a rigorous preprocessing and feature extraction pipeline was implemented using open source platform PyRadiomics (version 3.0.1) (23, 24). All segmented EAT volumes were first subjected to a standardized resampling and discretization process. The image voxels were resampled to an isotropic spacing of 2.0 × 2.0 × 2.0 mm^3^ using B-spline interpolation. Prior to resampling, intensity normalization was applied. A fixed bin width of 25 was used for gray-level discretization to ensure scale-invariant texture analysis. This method is based on the widely accepted recommendations in the field of radiomics, aiming to ensure the standardization of the gray scale for all cases, thereby reducing the bias caused by differences in scanning parameters (25). A total of 111 radiomics features were extracted from each patient's EAT segmentation to quantify its original imaging phenotype. These features were categorized into three classes: (1) 19 first-order features, summarizing the global intensity distribution; (2) 17 shape features, describing three-dimensional geometric properties; and (3) 75 texture features, capturing spatial intensity variation and heterogeneity. The texture features comprised 24 gray-level co-occurrence matrix (GLCM), 16 gray-level run-length matrix (GLRLM), 16 gray-level size zone matrix (GLSZM), 14 gray-level dependence matrix (GLDM), and 5 neighboring gray-tone difference matrix (NGTDM) features.

### 2.5 Features selection

The correlation clustering diagram of the radiomic features is shown in Supplementary Figure S1, where it can be observed that most of the radiomic features exhibit correlation and redundancy. This redundancy may introduce multicollinearity, which interferes with model training and reduces its generalization ability. In order to screen out the most discriminative feature subset to construct a robust prediction model, a feature importance assessment method based on random forest (RF) was used for feature selection. The principle of using RF for feature selection mainly lies in its ability to quantify the contribution of each feature to the model's prediction. The core idea is that if a feature is more useful for predicting the target variable, then when this feature is used in the model, the improvement in the model's performance should be more significant. It is precisely by leveraging its ability to robustly and efficiently identify key radiological biomarkers and clinical indicators most relevant to AF recurrence from high-dimensional radiomics and clinical features that the model's interpretability and generalization potential are enhanced. To ensure the robustness of the feature selection process and to prevent data leakage that could optimistically bias model performance estimates, all steps were embedded within a nested cross validation framework. In this study, the algorithm calculated and compared the contribution scores of all candidate features. Finally, according to the model performance, 22 key clinical features and 13 radiomics features with the highest discriminative value were retained. Furthermore, to construct a predictive model that extends beyond a single information domain, this study integrated clinical variables with radiomic features and again employed the RF algorithm to evaluate feature importance and identify key biomarkers within the fused high-dimensional feature space. A total of 34 features were finally selected into the fusion model. This process effectively reduced the data dimensionality and improved the clinical interpretability and generalization performance of the model.

### 2.6 Prediction model

To build robust and interpretable predictive models, and to comprehensively evaluate the performance of different algorithms in predicting AF recurrence, this study systematically selected six representative machine learning algorithms with distinct underlying principles: extreme gradient boosting (XGBoost), RF, Bayesian, K-Nearest Neighbors (KNN), Support Vector Machine (SVM), and Logistic Regression. The selection of this model was based on rigorous methodological considerations to ensure the robustness of the conclusions by covering different modeling paradigms. XGBoost and RF can effectively capture complex feature interactions. Logistic regression and Bayesian provide interpretable probabilistic output frameworks. KNN is used for prediction based on the local structure of the data. SVM is good at dealing with nonlinear problems. This diversification strategy helps the system to identify the optimal algorithm for this clinical prediction task. Before model training, to eliminate the impact of dimensions among different features and make the model more stable and faster converging during the training process, all data were standardized using the z-score method, transforming the data distribution into a standard normal distribution. All models were trained and evaluated under a 10-fold cross validation framework to ensure unbiased performance evaluation. The model was applied to the processed test set to generate predictions. All reported performance metrics (AUC, accuracy, sensitivity, specificity, F1-score) represent the mean ± standard deviation calculated from the predictions across all 10 outer-loop test folds. This rigorous pipeline guarantees an unbiased estimate of model generalizability by preventing any leakage of information from the test sets into the feature selection or model development stages. To comprehensively assess the predictive performance and clinical applicability of our models, we performed several additional analyses beyond conventional metrics. The clinical net benefit of each model was quantified using decision curve analysis (DCA), which assesses model utility across a range of threshold probabilities. Furthermore, to ensure that the predicted probabilities reliably reflected the actual risk, we further applied sigmoid calibration using CalibratedClassifierCV with 5-fold cross validation. Calibration performance was evaluated visually through calibration curves and quantitatively using the Brier score, a proper scoring rule in which lower values indicate better calibration.

### 2.7 Statistical analysis

The DSC was used to measure the effect of EAT image segmentation, and PSM was employed to control potential confounding variables. Categorical variables are presented as numbers (n) and percentages (%). Continuous variables are expressed as either mean ± standard deviation or median with interquartile range (IQR), based on their distribution. The 95% confidence interval (CI) was applied to assess statistical uncertainty, and the final quantitative results of the model were evaluated using the AUC, accuracy, sensitivity, specificity, and F1 score to assess the predictive performance of AF recurrence after PVI.

## 3 Result

### 3.1 Baseline information

After PSM score matching, 302 patients were finally included in the analysis. As shown in Table 1, the SMD for all covariates after matching were less than 0.1, indicating that baseline characteristics were strictly balanced between the groups. After matching, the average age of the case group was 61.83 years, of which 32.5% were women and 67.5% were men. The mean temperature in this group was 36.43 ℃, and the mean respiratory rate was 18.7 beats per minute. In the body mass index (BMI) subgroup, 49% were overweight (BMI: 23.0–27.5) and 33.8% were obese (BMI > 27.5). In terms of lifestyle habits (smoking, alcohol), medical conditions (hypertension, diabetes), and previous surgical history, the distributions were balanced between the case and control groups.

**Table 1:** Baseline characteristics of the eligible (before-match) and matched cohorts.

Characteristics	Eligible cohorts			Matched cohorts		
	Case group	Control group	SMD	Case group	Control group	SMD
Total	160	746		151	151	
Sex			0.15			0.014
Female (%)	51 (31.9%)	291 (39.0%)		49 (32.5%)	48 (31.8%)	
Male (%)	109 (68.1%)	455 (61.0%)		102 (67.5%)	103 (68.2%)	
Age [mean (SD)]	61.35 (10.97)	62.91 (11.27)	0.141	61.83 (10.75)	60.97 (11.37)	0.078
BMI			0.2			0.079
Normalweight: 18.5–23.0	26 (16.2%)	170 (22.8%)		25 (16.6%)	27 (17.9%)	
Overweight: 23.0–27.5	75 (46.9%)	343 (46.0%)		74 (49.0%)	71 (47.0%)	
Obese: >27.5	58 (36.2%)	223 (29.9%)		51 (33.8%)	51 (33.8%)	
Underweight: <18.5	1 (0.6%)	10 (1.3%)		1 (0.7%)	2 (1.3%)	
Body Temperature	36.43 (0.19)	36.46 (0.22)	0.155	36.43 (0.18)	36.43 (0.16)	0.023
Respiratory Rate[mean (SD)]	18.71 (1.07)	18.70 (1.10)	0.009	18.70 (1.07)	18.81 (1.11)	0.099
Smoking			0.063			0.061
Yes (%)	41 (25.6%)	171 (22.9%)		40 (26.5%)	36 (23.8%)	
No (%)	119 (74.4%)	575 (77.1%)		111 (73.5%)	115 (76.2%)	
Drinking			0.082			0.016
Yes (%)	33 (20.6%)	130 (17.4%)		32 (21.2%)	33 (21.9%)	
No (%)	127 (79.4%)	616 (82.6%)		119 (78.8%)	118 (78.1%)	
Diabetes			0.041			0.036
Yes (%)	24 (15.0%)	123 (16.5%)		24 (15.9%)	26 (17.2%)	
No (%)	136 (85.0%)	623 (83.5%)		127 (84.1%)	125 (82.8%)	
Hypertension			0.145			0.013
Yes (%)	78 (48.8%)	310 (41.6%)		71 (47.0%)	70 (46.4%)	
No (%)	82 (51.3%)	436 (58.4%)		80 (53.0%)	81 (53.6%)	
Operation History			0.178			0.067
Yes (%)	84 (52.5%)	457 (61.3%)		82 (54.3%)	87 (57.6%)	
No (%)	76 (47.5%)	289 (38.7%)		69 (45.7%)	64 (42.4%)	

### 3.2 Deep learning model for image segmentation

Despite the high anatomical variability of EAT in CTA images, the SwinUNETR model demonstrated robust and accurate segmentation performance. Quantitative evaluation on the independent validation set demonstrated a DSC of 0.87 ± 0.04 and HD of 2.83 ± 0.2, respectively. Representative visual results of the segmentation are provided in Supplementary Figure S2, confirming the model's effectiveness in delineating EAT boundaries. The training process was monitored through the loss and DSC curves. Optimal performance was achieved at 6,100 epochs, corresponding to the highest DSC on the validation set, with both metrics subsequently stabilizing, indicating convergence. The resulting high-quality segmentations provided the precise ROI necessary for subsequent radiomics feature extraction from the EAT.

### 3.3 Model prediction

**Figure 3:**
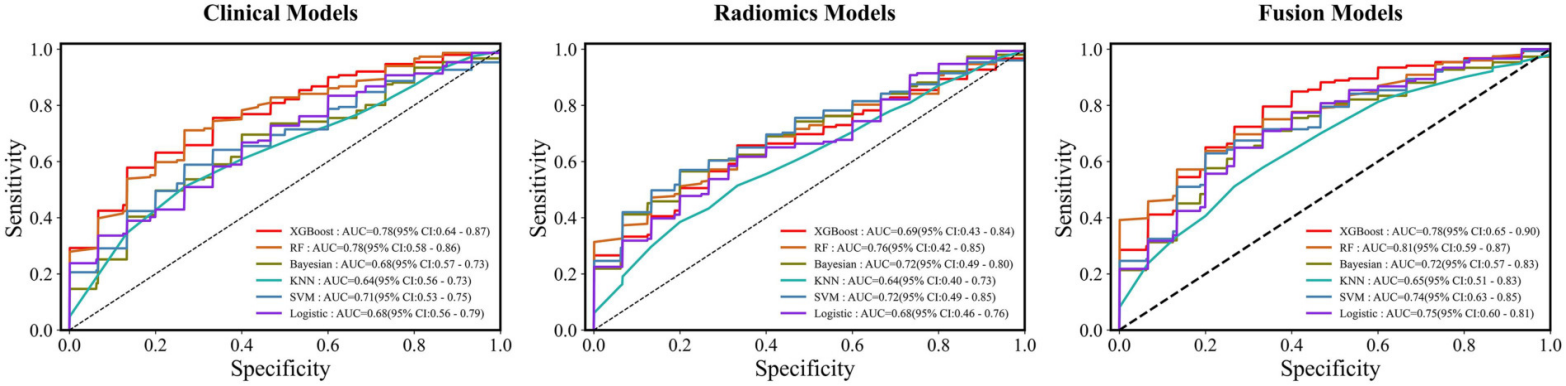
ROC curves of the clinical, radiomics, and fusion models for predicting AF recurrence after PVI. **(A)** clinical prediction model. **(B)** radiomics prediction model. **(C)** clinical and radiomics fusion model.

Following feature selection, 22 key clinical indicators and 13 radiomics features were retained for subsequent predictive modeling. The selected features, along with their importance scores from RF, are detailed in Supplementary Table S2 and Supplementary Table S3, respectively. The features of the fusion model are shown in Supplementary Table S4. The performance of machine learning algorithms was evaluated across three distinct model types: the clinical model (Table 2), the radiomics model (Table 3), and the integrated clinical-radiomics model (Table 4). Evaluation metrics included accuracy, sensitivity, specificity, F1-score, and the AUC. Figure 3 presents the receiver operating characteristic (ROC) curves for the three models: the clinical model (A), the radiomics model (B), and the fusion model (C).

**Table 2:** Performance metrics of clinical prediction models.

Model	Metric (95% CI)				
	Accuracy	Sensitivity	Specificity	F1	AUC
XGBoost	0.72 (0.58–0.79)	0.71 (0.55–0.85)	0.70 (0.55–0.85)	0.70 (0.56–0.80)	0.78 (0.64–0.87)
RF	0.70 (0.56–0.78)	0.70 (0.50–0.85)	0.70 (0.55–0.79)	0.69 (0.53–0.80)	0.78 (0.58–0.86)
Bayesian	0.66 (0.57–0.76)	0.53 (0.42–0.73)	0.80 (0.51–0.85)	0.61 (0.50–0.75)	0.68 (0.57–0.73)
KNN	0.57 (0.47–0.71)	0.73 (0.67–0.85)	0.35 (0.22–0.70)	0.63 (0.58–0.72)	0.64 (0.56–0.73)
SVM	0.60 (0.47–0.69)	0.32 (0.15–0.45)	0.87 (0.63–1.0)	0.43 (0.23–0.56)	0.71 (0.53–0.75)
Logistic	0.60 (0.52–0.69)	0.63 (0.47–0.81)	0.57 (0.42–0.73)	0.62 (0.50–0.70)	0.68(0.56–0.79)

**Table 3:** Performance metrics of radiomics prediction models.

Model	Metric (95% CI)				
	Accuracy	Sensitivity	Specificity	F1	AUC
XGBoost	0.67 (0.47–0.81)	0.67 (0.51–0.85)	0.67 (0.35–0.89)	0.68 (0.50–0.80)	0.69 (0.43–0.84)
RF	0.65 (0.44–0.79)	0.67 (0.35–0.73)	0.67 (0.50–0.90)	0.67 (0.39–0.76)	0.76 (0.42–0.85)
Bayesian	0.67 (0.47–0.79)	0.61 (0.42–0.77)	0.80 (0.43–0.87)	0.65 (0.47–0.78)	0.72 (0.49–0.80)
KNN	0.58 (0.39–0.69)	0.60 (0.36–0.72)	0.53 (0.30–0.85)	0.58 (0.38–0.66)	0.64 (0.40–0.73)
SVM	0.65 (0.57–0.74)	0.47 (0.33–0.65)	0.87 (0.68–0.98)	0.57 (0.48–0.71)	0.72 (0.49–0.85)
Logistic	0.60 (0.44–0.72)	0.58 (0.48–0.85)	0.53 (0.35–0.78)	0.60 (0.47–0.75)	0.68(0.46–0.76)

**Table 4:** Performance metrics of fusion prediction models.

Model	Metric (95% CI)				
	Accuracy	Sensitivity	Specificity	F1	AUC
XGBoost	0.73 (0.60–0.80)	0.77 (0.55–0.87)	0.70 (0.55–0.79)	0.75 (0.58–0.81)	0.78 (0.65–0.90)
RF	0.73 (0.51–0.80)	0.73 (0.46–0.85)	0.73 (0.56–0.85)	0.73 (0.49–0.81)	0.81 (0.59–0.87)
Bayesian	0.70 (0.55–0.76)	0.65 (0.43–0.73)	0.73 (0.56–0.87)	0.67 (0.52–0.74)	0.72 (0.57–0.83)
KNN	0.62 (0.49–0.76)	0.80 (0.68–0.87)	0.40 (0.23–0.72)	0.67 (0.59–0.77)	0.65 (0.51–0.83)
SVM	0.63 (0.57–0.72)	0.33 (0.28–0.53)	0.87 (0.80–0.98)	0.47 (0.42–0.65)	0.74 (0.63–0.85)
Logistic	0.70 (0.56–0.77)	0.73 (0.50–0.85)	0.67 (0.55–0.78)	0.71 (0.53–0.78)	0.75(0.60–0.81)

**Figure 4:**
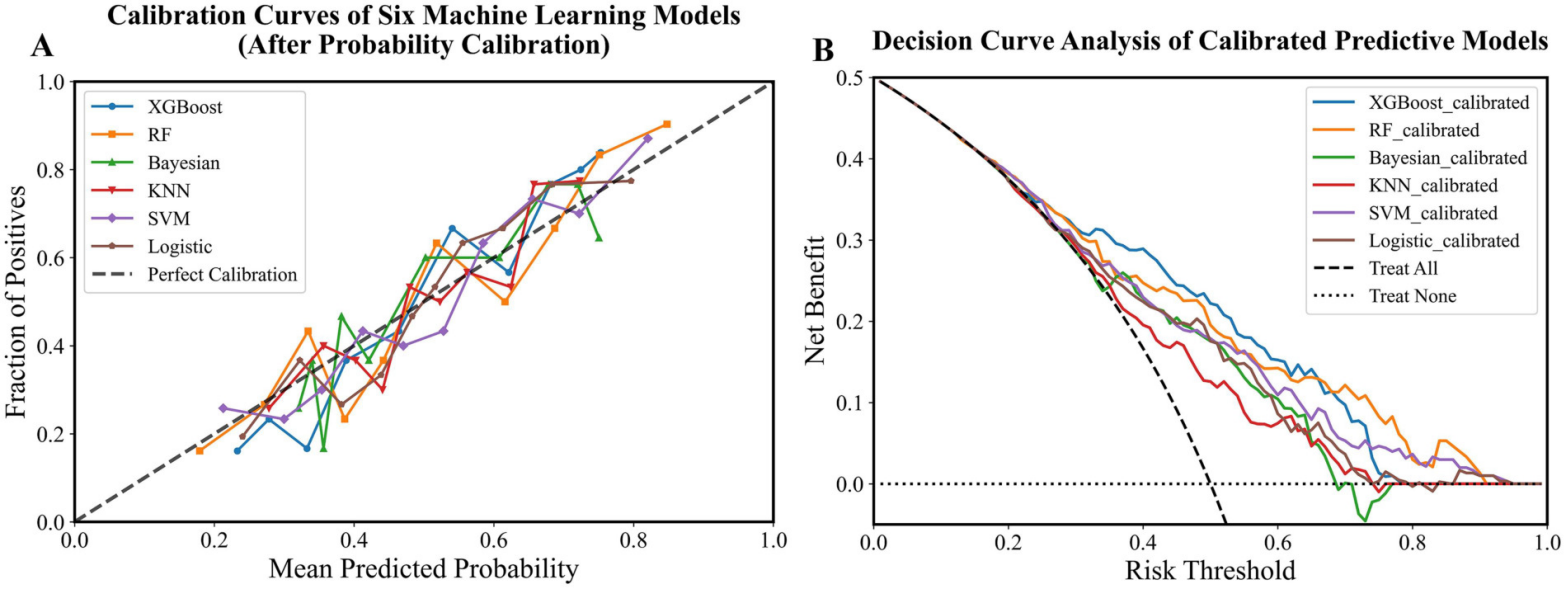
Model calibration and DCA. **(A)** Calibration curves of the six machine learning models. **(B)** DCA of the corresponding models, evaluating their clinical net benefit across varying risk thresholds.

To translate this discriminative ability into clinical value, DCA analysis was performed, and the DCA curve is shown in Figure 4B. At a clinically relevant decision threshold probability of 0.5, all calibrated models provided a positive net benefit, indicating their utility over the treat-none strategy. The XGBoost-based fusion model yielded the highest net benefit of 0.222, followed by the RF model (Net Benefit = 0.195). This translates to 22 more net beneficial interventions per 100 patients when using the XGBoost model for clinical decision-making at this threshold. The quantified net benefit of the calibrated models at a threshold probability of 0.5 is shown in Supplementary Table S5. The calibration performance of all models was significantly improved after sigmoid calibration. Post-calibration, the predicted probability ranges became more constrained and realistic, with means closely aligning with the overall event rate of 0.5. The calibration curves for the six machine learning models after probability calibration are presented in Figure 4A. The probability statistics and Brier scores of the calibrated models are presented in Supplementary Table S6. The Brier score was lowest for the XGBoost model (0.194), confirming its superior probability accuracy. All calibrated models achieved Brier scores below 0.23, indicating a satisfactory level of calibration for clinical interpretation.

## 4 Discussion

A model for predicting AF recurrence was developed and validated using pre-procedural data from patients undergoing PVI. To mitigate potential confounding biases, PSM was performed on key covariates, achieving a well-balanced cohort for analysis. Given the important role of EAT in the occurrence and development of AF, we specifically included the radiomics features and clinical variables. Accurate EAT segmentation is fundamental for reliable feature extraction. Therefore, we implemented a SwinUNETR model, trained via transfer learning on a set of 50 meticulously annotated CT scans. The model demonstrated robust performance, achieving a DSC of 0.87 on the validation set, and provided precise segmentations suitable for high-throughput radiomics analysis.

This study systematically evaluated the performance of six machine learning models in predicting AF recurrence after PVI. As shown in Tables 2, 3, and 4, the fusion model demonstrated the best and most robust overall predictive performance. Among them, XGBoost and RF achieved the highest discriminative efficacy within the fusion model, with AUC values of 0.78 (95% CI: 0.65–0.90) and 0.81 (95% CI: 0.59–0.87), respectively. These models also maintained balanced performance across other metrics, indicating that ensemble learning methods can effectively integrate multi-source information to enhance the overall predictive capability of the model. It is noteworthy that the predictive performance of the radiomics model (Table 3) was generally lower than that of the clinical model (Table 2) and the fusion model. This suggests that clinical prior knowledge still holds significant independent predictive value within the current feature framework. The addition of radiomic features further optimized model performance in the fusion model, highlighting their complementary role as incremental information. Models with different modeling principles show distinct characteristics: for instance, KNN showed high sensitivity but low specificity across all model types, reflecting its reliance on local data structure; SVM demonstrated high specificity but insufficient sensitivity, which may be related to sample size and the tuning of nonlinear kernel functions. The results of this study suggest that integrating clinical and radiomic information, along with ensemble learning algorithms such as XGBoost or RF, may lead to prediction models with improved performance and enhanced generalization ability. This provides a feasible computational approach for individualized risk assessment of AF recurrence after PVI. The applicability of the models in clinical decision-making was further supported by DCA and calibration assessment. The DCA results demonstrated that the calibrated XGBoost model achieved the highest net benefit at the commonly used decision threshold of 0.5, indicating superior clinical utility in balancing false positives and false negatives. Moreover, the calibration procedure substantially improved the probability output distributions of all models, bringing them closer to the observed event rates. This improvement was particularly notable for models that originally exhibited wide or markedly biased probability ranges. Based on the Brier scores, XGBoost and RF demonstrated the best performance, indicating superior accuracy and stability in probability estimation compared with the other models. This finding is consistent with previous evaluations using AUC and accuracy, further reinforcing the advantage of tree-based models for this prediction task. It is noteworthy that although calibration improves the reliability of predicted probabilities, it may also compress the probability range. Future studies may incorporate additional metrics such as net reclassification improvement (NRI) to more comprehensively evaluate the contribution of these models to clinical risk stratification.

The feature selection process yielded clinical and radiomic biomarkers that align coherently with the known pathophysiology of AF recurrence, particularly through the lens of EAT activity. Among the top-ranked clinical indicators (Supplementary Table S2), Elevated NT-proBNP levels reflect abnormalities in heart function, such as increased ventricular wall tension or pressure, enlargement of the left atrium, and cardiac fibrosis (26–28). Its prominence underscores the role of underlying cardiac structural and functional impairment in promoting arrhythmic recurrence. Additionally, indicators of metabolic dysregulation (HbA1c, LDL-C) and systemic inflammation (GLO, FDP) were retained, reflecting the systemic pro-inflammatory and pro-fibrotic milieu that facilitates atrial remodeling (29–32). The radiomic features (Supplementary Table S3) further quantify the local impact of EAT. High-ranking texture features such as gray level non-uniformity (GLSZM GLNN) and gray level variance (GLSZM GLV) directly reflect tissue heterogeneity, which may correspond histologically to varying degrees of adipocyte size, inflammatory cell infiltration, and fibrosis within EAT. Similarly, GLCM cluster shade (GLCM CS) and shape-based features (spherical disproportion, compactness) capture the spatial asymmetry and geometric complexity of EAT. Potentially linked to invasive growth patterns and paracrine interactions with the atrial myocardium. In the fusion model (Supplementary Table S4), with several from each domain maintaining significant weight. Notably, NT-proBNP, blood pressure markers (SBP), and texture heterogeneity features (GLSZM GLNN, GLCM CS) persisted as key contributors. This finding indicates that while systemic factors establish the foundational pathological environment for AF recurrence, EAT imaging biomarkers reflect localized atrial adipose involvement. Furthermore, the retained morphological features suggest that not only texture but also the anatomical distribution and contour irregularity of EAT may jointly influence atrial electrophysiology.

Beyond the established paracrine and inflammatory pathways, our radiomic assessment of EAT heterogeneity may also be capturing a phenotype relevant to its physical influence on ablation efficacy. Adipose tissue possesses inherent thermal insulating properties (33). This property may be particularly relevant in the context of catheter ablation, where periatrial epicardial fat could attenuate energy transfer to the atrial myocardium. This thermoprotective effect might lead to insufficient lesion depth or contiguity, resulting in incomplete PVI and thereby elevating the risk of arrhythmia recurrence (34, 35). Thus, the EAT features identified in our model may serve a dual interpretive role: as biomarkers of local pathogenic activity and as indirect indicators of a physical environment less conducive to durable ablation lesion formation.

This mechanistic insight has direct implications for personalized procedural planning. The emergence of pulsed field ablation (PFA), which utilizes non-thermal, electroporation-based cell death with high myocardial selectivity, may offer a distinct advantage in this context (36–38). This allows it to overcome the thermal insulation effect of EAT, which often acts as a physical and thermal barrier, potentially impeding the transmurality of thermal lesions. By circumventing the energy-attenuating thermoprotective effect of EAT, PFA could potentially achieve more consistent and transmural lesions in patients with abundant or heterogenous periatrial fat, as quantified by radiomics. Therefore, the radiomic profiling of EAT developed in this study could evolve beyond a prognostic tool. In the future, It may aid in pre-procedural decision-making by identifying patients with a high risk EAT phenotype. These patients could derive greater benefit from PFA than from conventional thermal ablation, personalizing both risk assessment and therapeutic strategy.

Collectively, these selected features map coherently onto the biological narrative of AF recurrence: systemic metabolic and inflammatory signals create a permissive environment, while EAT, which is quantified via its radiomic phenotype, acts as a local effector of inflammation, fibrosis, and electrical disturbance. Our model thus operationalizes this mechanism by integrating circulating biomarkers with imaging signatures of adipose tissue activity, offering a multiscale perspective on post-PVI recurrence risk. It is important to note that our model is based on structural and textural information derived from CT. The incorporation of functional dynamic assessments, particularly from advanced echocardiography techniques such as left atrial strain and tissue doppler-derived indices, could provide a complementary layer of information regarding atrial mechanical and electrical substrate. Future multimodal studies that combine CT-based radiomics with echocardiographic functional parameters hold significant promise for building an even more comprehensive and physiologically nuanced risk prediction model for AF recurrence.

## 5 Limitations

This study has several limitations that should be acknowledged. First, the retrospective and single-center design of this study inherently limits the generalizability of our findings and may introduce selection bias. Although PSM was applied to balance key baseline characteristics and sensitivity analysis suggested the robustness of our core results, the exclusion of a substantial number of cases due to incomplete data may further affect the model's applicability to broader AF populations with heterogeneous data quality. Furthermore, the PSM design used in this study changed the event distribution of the cohort while optimizing the comparability between groups. Therefore, the model performance indicators and probability calibration are mainly suitable for the internal validation environment. Future multi-center, prospective studies, conducted under a predefined and standardized data collection protocol, are required to validate and confirm the universal utility of our model. Second, and specific to the imaging analysis pipeline, our deep learning model for EAT segmentation was trained on a manually annotated dataset of only 50 CT scans. While the use of a pre-trained SwinUNETR backbone and extensive data augmentation mitigated overfitting and yielded a clinically acceptable segmentation performance, the relatively small scale of this training set remains a constraint. This may limit the model's robustness when applied to CT images acquired with different protocols or to patients exhibiting atypical anatomical variations, potentially affecting the stability of downstream radiomics feature extraction. Third, the radiomics features were derived from pre-operative CT scans. The biological interpretation of the identified textural signatures remains speculative due to the lack of direct histopathological correlation and the potential influence of variability in imaging acquisition parameters. Future studies integrating intra-operative tissue sampling could help bridge this gap between imaging phenotypes and molecular pathophysiology. Moreover, while our CT-based radiomics model provides novel structural insights, its predictive scope could be further expanded. The present work did not evaluate parameters from advanced echocardiography, such as atrial strain or tissue Doppler indices, which are established markers of atrial functional substrate. A truly multimodal assessment, combining the strengths of CT anatomy, tissue texture, and echocardiographic function, is likely to yield a superior, holistic predictor of post-ablation outcome, and should be a priority for future research. Finally, while the fusion model demonstrated improved predictive performance, its “black-box” nature poses a challenge for clinical interpretation and trust. The development and integration of explainable AI techniques to elucidate the contribution of decisive image regions will be crucial for fostering clinical adoption and translating the model into a practical decision-support tool.

## 6 Conclusion

This study developed and validated a machine learning model integrating pre-procedural clinical features and EAT radiomics to predict AF recurrence after PVI. The results demonstrate that the fusion model, particularly when built upon ensemble algorithms such as XGBoost or RF, achieved the optimal predictive performance and robustness. Feature analysis further revealed the synergistic mechanism of systemic metabolic inflammation environment and local adipose tissue heterogeneity in AF recurrence, providing a multi-scale perspective for understanding its pathophysiology. This model provides a potential computational tool for individualized recurrence risk stratification, and its clinical application value needs to be further verified by prospective studies.

## Data Availability

The raw data supporting the conclusions of this article will be made available by the authors, without undue reservation.
