# Supplementary material for: Epicardial adipose tissue radiomic features from pre-procedural CT to predict atrial fibrillation recurrence after catheter ablation for pulmonary vein isolation
Source: Front Cardiovasc Med. 2026 Feb 26;13:1765419. doi: 10.3389/fcvm.2026.1765419 (PMC12979507; doi:10.3389/fcvm.2026.1765419)

**Supplement**

**Supplementary Table S1.** Comparison of baseline characteristics between the primary cohort and the prematching cohort.

| **Characteristics** | **Primary cohort** | | **Prematching cohort** | |
| --- | --- | --- | --- | --- |
|  | **Case group** | **Control group** | **Case group** | **Control group** |
| Total | 160 | 1391 | 160 | 746 |
| Sex |  |  |  |  |
| Female (%) | 51 (31.9) | 520 (37.4) | 51 (31.9) | 291 (39.0) |
| Male (%) | 109 (68.1) | 871 (62.6) | 109 (68.1) | 455 (61.0) |
| Age (mean (SD)) | 61.35 (10.97) | 62.55 (11.74) | 61.35 (10.97) | 62.91 (11.27) |
| BMI |  |  |  |  |
| Normalweight:18.5-23.0 | 26 (16.2) | 280 (20.1) | 26 (16.2) | 170 (22.8) |
| Overweight:23.0-27.5 | 75 (46.9) | 672 (48.3) | 75 (46.9) | 343 (46.0) |
| Obese:>27.5 | 58 (36.2) | 427 (30.7) | 58 (36.2) | 223 (29.9) |
| Underweight:<18.5 | 1 ( 0.6) | 12 ( 0.9) | 1 ( 0.6) | 10 ( 1.3) |
| Body Temperature | 36.43 (0.19) | 36.46 (0.21) | 36.43 (0.19) | 36.46 (0.22) |
| Respiratory Rate(mean (SD)) | 18.71 (1.07) | 18.73 (1.10) | 18.71 (1.07) | 18.70 (1.10) |
| Smoking |  |  |  |  |
| Yes (%) | 41 (25.6) | 320 (23.0) | 41 (25.6) | 171 (22.9) |
| No (%) | 119 (74.4) | 1071 (77.0) | 119 (74.4) | 575 (77.1) |
| Drinking |  |  |  |  |
| Yes (%) | 33 (20.6) | 241 (17.3) | 33 (20.6) | 130 (17.4) |
| No (%) | 127 (79.4) | 1150 (82.7) | 127 (79.4) | 616 (82.6) |
| Diabetes |  |  |  |  |
| Yes (%) | 24 (15.0) | 224 (16.1) | 24 (15.0) | 123 (16.5) |
| No (%) | 136 (85.0) | 1167 (83.9) | 136 (85.0) | 623 (83.5) |
| Hypertension |  |  |  |  |
| Yes (%) | 78 (48.8) | 585 (42.1) | 78 (48.8) | 310 (41.6) |
| No (%) | 82 (51.2) | 806 (57.9) | 82 (51.2) | 436 (58.4) |
| Operation History |  |  |  |  |
| Yes (%) | 84 (52.5) | 815 (58.6) | 84 (52.5) | 457 (61.3) |
| No (%) | 76 (47.5) | 576 (41.4) | 76 (47.5) | 289 (38.7) |

**Supplementary Table S2.** Clinical indicators selected by random forest and variable coefficients.

| **No.** | **Clinical Indicators** | **Abbreviation** | **Coefficient** |
| --- | --- | --- | --- |
| 1 | N-Terminal Pro-Brain Natriuretic Peptide | NT-proBNP | 0.0502 |
| 2 | Mean Corpuscular Hemoglobin Concentration | MCHC | 0.0221 |
| 3 | Pulse | pulse | 0.0213 |
| 4 | Heart Rate | HR | 0.0201 |
| 5 | Systolic Blood Pressure | SBP | 0.0193 |
| 6 | Mean Corpuscular Hemoglobin | MCH | 0.0182 |
| 7 | P | P | 0.0181 |
| 8 | Low-Density Lipoprotein Cholesterol | LDL-C | 0.0180 |
| 9 | Glycosylated Hemoglobin A1c | HbA1c | 0.0178 |
| 10 | Fibrinogen Degra-Dation Products | FDP | 0.0172 |
| 11 | Diastolic Blood Pressure | DBP | 0.0171 |
| 12 | Total Thyroxine | TT4 | 0.0170 |
| 13 | Aspartate Transferase | AST | 0.0165 |
| 14 | Globulin | GLO | 0.0164 |
| 15 | Total Bile Acid | TBA | 0.0163 |
| 16 | Ggamma Glutamyl Transferase | GGT | 0.0163 |
| 17 | Indirect Bilirubin | IBIL | 0.0162 |
| 18 | Total Bilirubin | TBIL | 0.0161 |
| 19 | Creatine Kinase | CK | 0.0159 |
| 20 | Free Thyroxine | FT4 | 0.0155 |
| 21 | Lipoprotein a | LPa | 0.0151 |
| 22 | Serum Total Protein | TP | 0.0149 |

**Supplementary Table S3.** Radiomics features selected by random forest and variable coefficients.

| **No.** | **Radiomics Features** | **Abbreviation** | **Coefficient** |
| --- | --- | --- | --- |
| 1 | GLSZM Gray Level Non-Uniformity Normalized | **GLSZM** GLNN | 0.0196 |
| 2 | GLSZM Gray Level Variance | **GLSZM GLV** | 0.0172 |
| 3 | GLCM Cluster Shade | **GLCM** CS | 0.0162 |
| 4 | Shape Spherical Disproportion | - | 0.0161 |
| 5 | Firstorder Maximum | - | 0.0149 |
| 6 | **GLCM** Maximum Probability | **GLCM** MP | 0.0145 |
| 7 | Glszm **Gray Level Non-Uniformity** | **GLN** | 0.0142 |
| 8 | Firstorder Skewness | - | 0.0140 |
| 9 | Shape Compactness1 | - | 0.0137 |
| 10 | Shape Compactness2 | - | 0.0137 |
| 11 | Shape **Maximum 2D diameter (Row)** | - | 0.0133 |
| 12 | Shape Sphericity | - | 0.0128 |
| 13 | Glcm **Difference Variance** | - | 0.0127 |

**Supplementary Table S4.** Fusion model features and variable coefficients.

| **No.** | **Fusion Features** | **Abbreviation** | **Coefficient** |
| --- | --- | --- | --- |
| 1 | N-Terminal Pro-Brain Natriuretic Peptide | NT-ProBNP | 0.0264 |
| 2 | Systolic Blood Pressure | SBP | 0.0114 |
| 3 | **GLSZM Gray Level Non-Uniformity Normalized** | **GLSZM** GLNN | 0.0105 |
| 4 | Low-Density Lipoprotein Cholesterol | LDL-C | 0.0102 |
| 5 | **GLCM Cluster Shade** | **GLCM** CS | 0.0100 |
| 6 | Pulse | - | 0.0100 |
| 7 | Heart Rate | HR | 0.0099 |
| 8 | **GLSZM Gray Level Variance** | **GLSZM GLV** | 0.0098 |
| 9 | P | P | 0.0097 |
| 10 | Total Cholesterol | TC | 0.0091 |
| 11 | Globulin | GLO | 0.0091 |
| 12 | Shape Compactness1 | - | 0.0090 |
| 13 | Prothrombin Time International Normalized Ratio | PT-INR | 0.0090 |
| 14 | Shape Sphericity | - | 0.0088 |
| 15 | Total Bilirubin | TBIL | 0.0088 |
| 16 | Shape Compactness2 | - | 0.0086 |
| 17 | Firstorder Maximum | - | 0.0085 |
| 18 | Mean Corpuscular Hemoglobin | MCH | 0.0084 |
| 19 | Glcm Joint Energy | - | 0.0084 |
| 20 | Glcm Maximum Probability | - | 0.0083 |
| 21 | Fibrinogen Degra-Dation Products | FDP | 0.0082 |
| 22 | Aspartate Transferase | AST | 0.0082 |
| 23 | Glszm Gray Level Non Uniformity | **GLN** | 0.0082 |
| 24 | Mean Corpuscular Hemoglobin Concentration | MCHC | 0.0080 |
| 25 | Indirect Bilirubin | IBIL | 0.0078 |
| 26 | Glycosylated Hemoglobin A1c | HbA1c | 0.0078 |
| 27 | Creatine Kinase | CK | 0.0077 |
| 28 | Free Thyroxine | FT4 | 0.0077 |
| 29 | Shape Spherical Disproportion | - | 0.0076 |
| 30 | Thyroid Stimulating Hormone | TSH | 0.0075 |
| 31 | Total Bile Acid | TBA | 0.0075 |
| 32 | Total Thyroxine | TT4 | 0.0074 |
| 33 | Diastolic Blood Pressure | DBP | 0.0074 |
| 34 | Shape **Maximum 2D Diameter (Row)** | - | 0.0074 |

**Supplementary Table S5.** DCA showing the net benefit of calibrated models at a threshold probability of 0.5.

| **Calibrated Models** | **Net Benefit** |
| --- | --- |
| **XGBoost** | **0.222** |
| **RF** | **0.195** |
| **Bayesian** | **0.176** |
| **SVM** | **0.179** |
| **Logistic** | **0.176** |
| **KNN** | **0.126** |

**Supplementary Table S6.** Probability statistics and Brier scores of calibrated models.

| ****Model**** | ****Min**** | ****Max**** | ****Mean**** | ****Std**** | ****Pos. Mean**** | ****Neg. Mean**** | ****Brier Score**** |
| --- | --- | --- | --- | --- | --- | --- | --- |
| **XGBoost** | **0.203** | **0.781** | **0.502** | **0.182** | **0.591** | **0.412** | **0.194** |
| **RF** | **0.117** | **0.908** | **0.503** | **0.210** | **0.601** | **0.406** | **0.197** |
| **Bayesian** | **0.314** | **0.765** | **0.508** | **0.160** | **0.565** | **0.451** | **0.219** |
| **SVM** | **0.126** | **0.943** | **0.506** | **0.186** | **0.583** | **0.429** | **0.208** |
| **Logistic** | **0.147** | **0.947** | **0.503** | **0.163** | **0.564** | **0.442** | **0.216** |
| **KNN** | **0.167** | **0.753** | **0.505** | **0.136** | **0.546** | **0.464** | **0.228** |

**Supplementary Figure S1.** The correlation clustering diagram of the radiomic features.
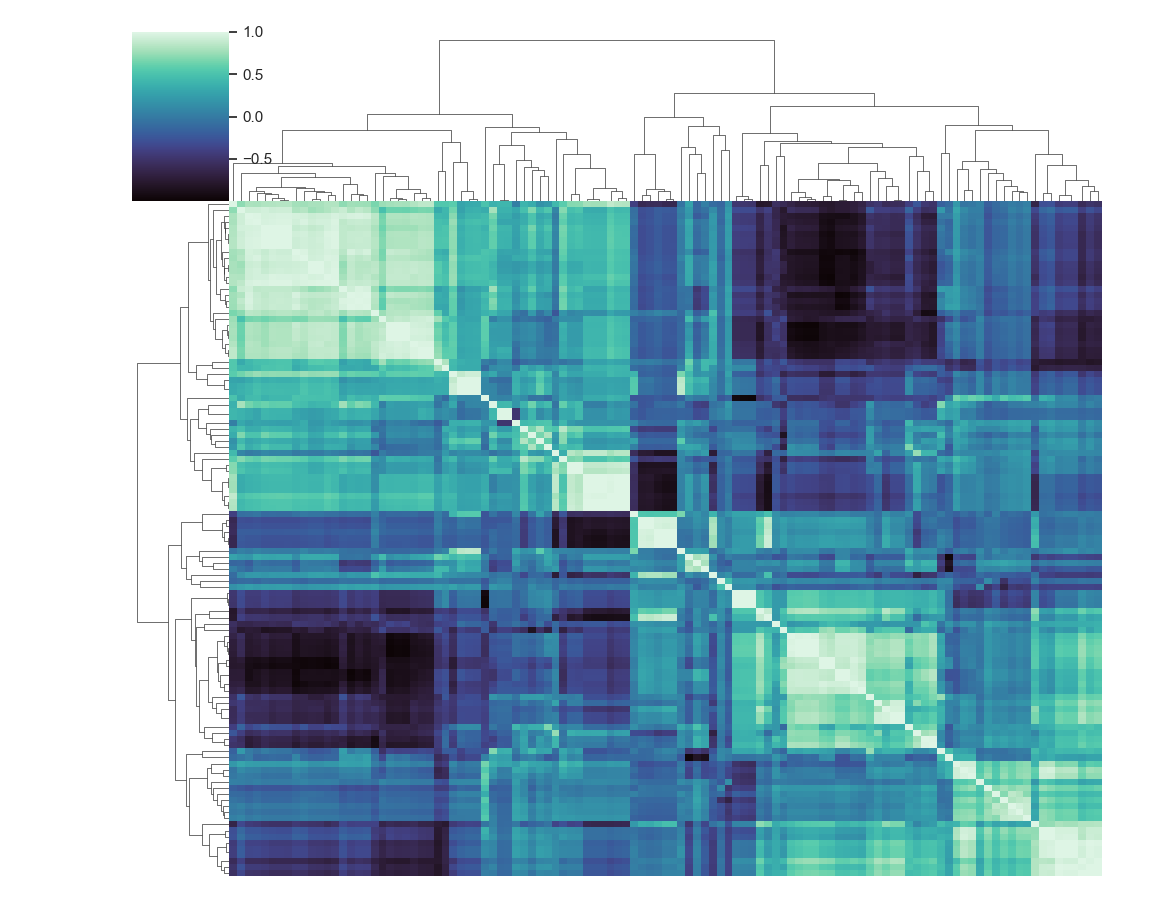


**Supplementary Figure S2.** Visualization of EAT segmentation results.


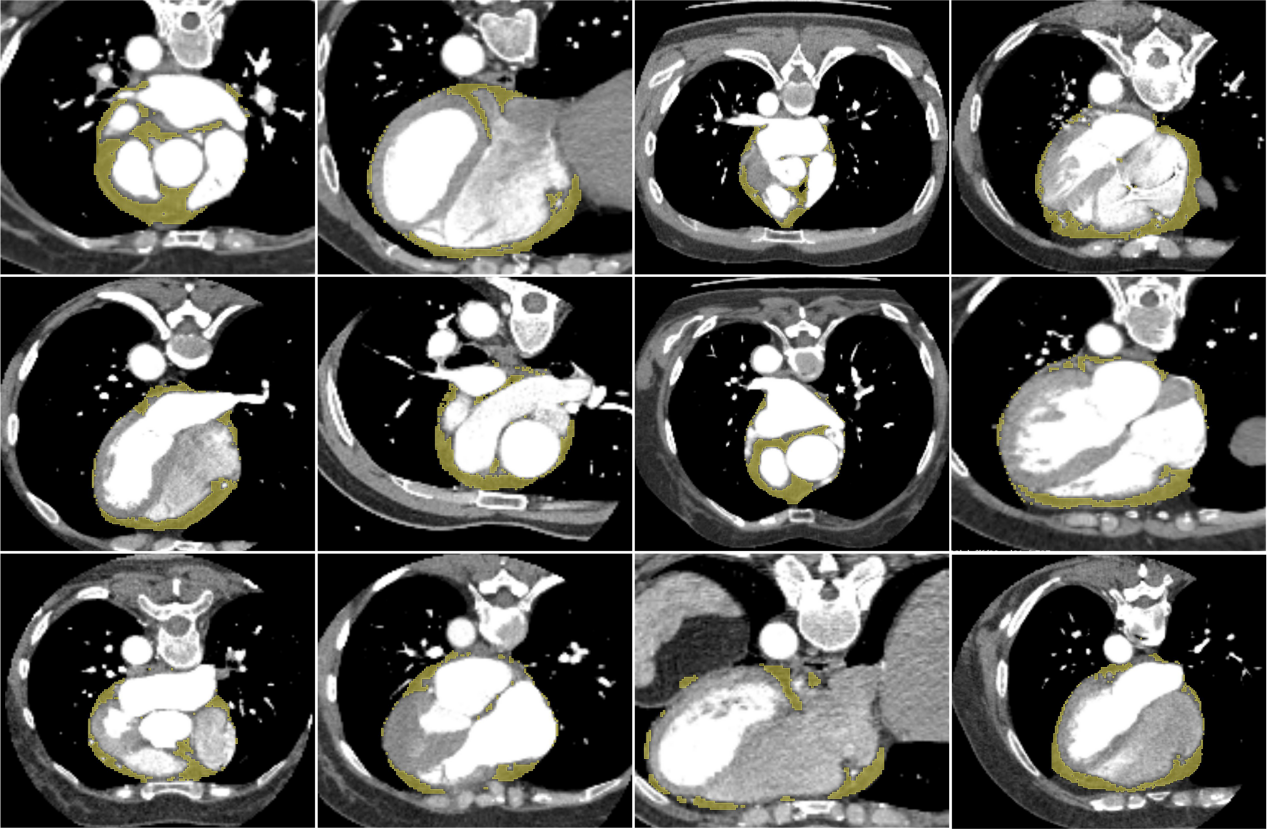

Supplement: Supplementary file 1 [file Datasheet1.docx]
